# Did the French pregnancy pictogram change antiseizure medication use during pregnancy?

**DOI:** 10.1002/epi.70163

**Published:** 2026-03-09

**Authors:** Mélanie Araujo, Christine Damase‐Michel, Marie Denuelle, Sébastien Boulogne, Laurane Delteil, Monica Saucedo, Catherine Deneux‐Tharaux, Isabelle Lacroix

**Affiliations:** ^1^ Faculté de Médecine de Toulouse Centre Hospitalier Universitaire de Toulouse, Institut National de la Santé et de la Recherche Médicale (INSERM) Unité Mixte de Recherche (UMR) 1295 Toulouse France; ^2^ Electrophysiologie Cérébrale, Épilepsie et Sommeil Hôpital Pierre Paul Riquet, CHU Purpan Toulouse France; ^3^ Service de Neurologie Fonctionnelle et d'épileptologie Groupement Hospitalier Est–Hôpital Pierre Wertheimer Bron France; ^4^ Institut National de la Santé et de la Recherche Médicale (INSERM), Institut National de Recherche pour l'Agriculture, l'Alimentation et l'Environnement (INRAE), Center for Research in Epidemiology and Statistics, Obstetrical Perinatal and Pediatric Life Course Epidemiology Université Paris Cité, Université Paris Nord Paris France

**Keywords:** antiseizure medications, impact study, pictogram, pregnancy, SNDS database

## Abstract

**Objective:**

The aim of this study was to assess the impact of introducing a pregnancy pictogram on medication packaging on the prescription and dispensation of antiseizure medications and on the maternal and neonatal outcomes for women exposed to these medications.

**Methods:**

This is a national retrospective cohort study, based on the French National Health Data System, with a “before/after the introduction of the pictogram” design. Women aged between 15 and 55 years who had a pregnancy outcome between 2014 and 2017 (“before pictogram” period) and between 2018 and 2021 (“after pictogram” period) and who had received at least two antiseizure medication dispensations before their pregnancy were included. We compared the rates of antiseizure medication discontinuation and continuation during pregnancy, the average doses used, and maternal and neonatal outcomes between the two periods.

**Results:**

The rate of women who had received at least two dispensations of a medication indicated for epilepsy before their pregnancy remained stable between the two periods (.7%). There was a significant decrease in valproic acid prescriptions (5.4% vs. 1.3%) during pregnancy and, conversely, an increase in lamotrigine (29.9% vs. 31.5%) and levetiracetam (10.9% vs. 14.5%) prescriptions. Prescriptions by specialists such as neurologists increased significantly (22.8% vs. 28%) between the two periods. There was an increase of more than 2.7% in the continuation of antiseizure medication (37.6% vs. 40.3%, *p* < .0001) and conversely a decrease in the rate of women who stopped their antiseizure treatment before or during pregnancy in the “after pictogram” period (59.4% vs. 56.7%, *p* < .0001). Rates of maternal and neonatal outcomes remained similar between the two periods.

**Significance:**

The introduction of a pregnancy pictogram in France in 2017 was not associated with an increase in discontinuation of antiseizure medications, less adequate treatment, or poorer maternal or neonatal outcomes in pregnant women receiving these medications before pregnancy.

**Key points**

- The introduction of the pregnancy pictogram on medication packaging in France in 2017 was not associated with adverse trends in prescription and dispensation practices for antiseizure medications during pregnancy.
- Concerns that the pictogram would increase treatment discontinuation among pregnant women are therefore not supported by our results, as discontinuation rates did not rise.
- We observed a concomitant favorable shift toward greater use of lamotrigine and levetiracetam and a marked decline in valproate prescriptions.

## 1 INTRODUCTION

Antiseizure medications (ASMs) are prescribed to treat various chronic conditions, including epilepsy, neuropathic pain, bipolar disorder, anxiety, and migraine. Studies have reported that ASMs are prescribed for 1.2%–3% of women of childbearing age^1^ and .4%–.7% of pregnant women.^2^ For these women, particularly those with epilepsy, treatment should be adapted for the pregnancy as far as possible and often cannot be discontinued. Patients who discontinue their treatment are at high risk of decompensation of their condition and severe epileptic seizures, which may be fatal.^3^, ^4^ The risk of maternal death in women with epilepsy is 5–10 times higher than in women without epilepsy.^5^, ^6^, ^7^

In France, following the “valproic acid’ affair (victims of valproic acid use during pregnancy filed a complaint against a pharmaceutical firm and the health authorities), in 2017 the public authorities required pharmaceutical firms to include a specific pictogram on the outer packaging of medications that pose a risk during pregnancy. Two pictograms are available, one with the word “danger” and one with “prohibited” (Table 1). For medicolegal protection, and in the absence of a precise definition of “risk medications,” pharmaceutical firms have widely used this pictogram on their packaging. To date, approximately 10% of medications have clearly established teratogenic and/or fetotoxic effects in humans. In France today, approximately 60% of proprietary medicinal products have a “prohibited” or “danger” pictogram on their outer packaging.^8^ All ASMs have a pictogram on their packaging.

**TABLE 1:** Most commonly prescribed antiseizure medications during pregnancy among women who received these medications (whatever the indication) before pregnancy,^a^ by period (before and after pictogram); *N* = 29 080.

Antiseizure medication	"Before pictogram" period, *n* = 14 732	"After pictogram" period, *n* = 14 348	*p*
	Pregnant women, *n* (%)		
Lamotrigine	4402 (29.9%)	4523 (31.5%)	.**002**
Levetiracetam	1604 (10.9%)	2078 (14.5%)	**<.0001**
Clobazam	1032 (7.0%)	949 (6.6%)	.19
Pregabalin	817 (5.5%)	1036 (7.2%)	**<.0001**
Valproic acid	797 (5.4%)	193 (1.3%)	**<.0001**
Carbamazepine	533 (3.6%)	373 (2.6%)	**<.0001**
Topiramate	433 (2.9%)	339 (2.4%)	.**002**
Diazepam	290 (2.0%)	325 (2.3%)	.08
Gabapentin	290 (2.0%)	301 (2.1%)	.43
Oxcarbazepine	187 (1.3%)	188 (1.3%)	.76
Clonazepam	177 (1.2%)	128 (.9%)	.**01**
Lacosamide	111 (.8%)	163 (1.1%)	.**001**
Zonisamide	83 (.6%)	93 (.6%)	.35
Phenobarbital	86 (.6%)	40 (.3%)	**<.0001**
Eslicarbazepine	29 (.2%)	53 (.4%)	.**01**
Ethosuximide	17 (.1%)	9 (.1%)	–
Perampanel	16 (.1%)	51 (.4%)	**<.0001**
Midazolam	7 (.1%)	14 (.1%)	–
Phenytoin	3 (.02%)	3 (.02%)	–
Primidone	3 (.02%)	1 (.01%)	–
Vigabatrin	2 (.01%)	3 (.02%)	–
Stiripentol	1 (.01%)	0 (.0%)	–
Brivaracetam	0 (.0%)	2 (.01%)	–

*Note*: Boldface indicates statistical significance.

^a^ At least two dispensations in the year before the start of the pregnancy.

Several organizations and academic bodies (national academies of medicine and pharmacy, etc.) have warned of the potentially harmful effects of this new regulation.^9^, ^10^ In particular, there is a potential risk that women will stop any treatment, however essential it may be for their health, because of a worrying pictogram. In women with epilepsy, maintaining seizure control during pregnancy is a priority. Among available options, lamotrigine and levetiracetam are currently considered the most appropriate first‐line treatments during pregnancy^11^ due to their relatively favorable safety profiles for both mother and fetus. However, in France, the packaging of medications containing lamotrigine now carries a “danger” pictogram with the text “Do not use in pregnant women unless there is no therapeutic alternative.”

To our knowledge, no study has assessed the impact of this new pictogram on maternal health or on the course of pregnancy. Such an assessment seems necessary to determine whether the "danger" or "prohibited" pictograms have a harmful effect on the suitability of medications during pregnancy for women treated with ASMs, with potentially serious consequences for their health (disease complications and/or deleterious effects on pregnancy). Data from the French National Health Data System (SNDS) provides an opportunity to conduct this assessment on a national scale. This national database makes it possible to study the dispensation of medications reimbursed by health insurance before and during pregnancy, as well as pregnancy outcomes and complications.^12^

The aim of our project was to evaluate the impact of the pregnancy pictogram introduced in France in 2017 on the prescription and dispensation of ASMs during pregnancy, as well as on maternal and neonatal outcomes in women taking these medications before pregnancy.

## 2 MATERIALS AND METHODS

This is a national retrospective cohort study, with a "before/after" (the introduction of the pictogram) approach.

### 2.1 Data source

The data source used was the SNDS, a pseudonymized medicoadministrative database covering the entire French population and including all care reimbursed by health insurance.^12^ Created in 2016, the SNDS links data on medical care reimbursed by health insurance (SNIIRAM database), data on hospitalizations including medical diagnoses (PMSI database), and deaths (CépiDC database). In the SNDS, diagnoses were coded according to the 10th revision of the International Classification of Diseases (ICD‐10).

Pregnancies were identified using hospital stays related to deliveries (diagnosis codes starting with Z37 or childbirth procedures JQGD001‐005, JQGD007‐008, JQGD010, JQGD012‐013, and JQGA003‐005) and to births, present in the PMSI MCO (Medicine, Surgery, Obstetrics, and Odontology) tables of the SNDS. Pregnancies meeting the inclusion criteria and recorded in the SNDS between 2014 and 2021 were included. In France, more than 99% of births take place in hospitals.

### 2.2 Study population

**FIGURE 1:**
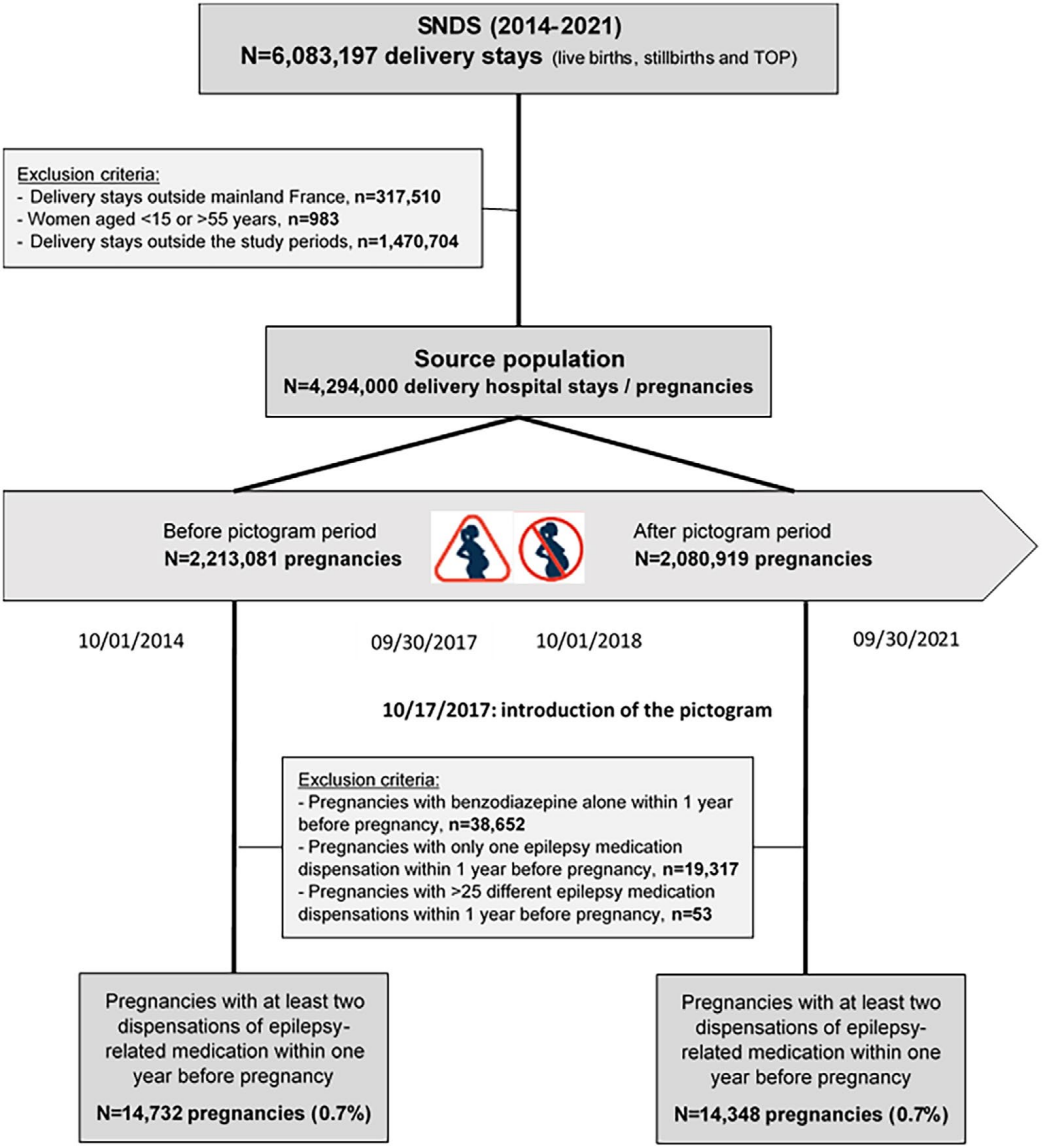
Flowchart. SNDS, French National Health Data System; TOP, total of perinatal deaths.

Our study population included all women aged 15 to 55 who had a pregnancy duration of 22 weeks of amenorrhea or more and who gave birth between October 1, 2014, and September 30, 2017 (the “pre‐pictogram” period) and between October 1, 2018, and September 30, 2021 (the “post‐pictogram” period). The pictogram was introduced in France on October 17, 2017. We further selected women who had received at least two reimbursements for the dispensation of medications indicated for epilepsy (these medications may have been prescribed for other indications) in the year preceding the date of their last menstrual period (LMP). The flowchart is shown in Figure 1.

Medications indicated for epilepsy were selected using the Anatomical Therapeutic Chemical (ATC) classification. The following ATC codes were used: N03A, antiepileptics; N05BA09, clobazam; N05BA01, diazepam; and N05CD08, midazolam. Women exposed to a benzodiazepine alone (diazepam, clobazam, midazolam and/or clonazepam) were excluded.

### 2.3 Statistical analysis

Continuous variables are presented as mean (±SD and range [minimum–maximum]). Student *t*‐test or the Mann–Whitney *U*‐test was used for comparisons. Categorical variables are expressed as number and percentage; chi‐squared test or Fisher exact test (if *n* ≤ 5) was used. A two‐tailed significance level of 5% was applied.

First, we described and compared the rates of women receiving medications indicated for epilepsy (whatever the indication), and among the receivers, the most commonly prescribed ASMs and the mean women's age, between the two periods (before and after the pictogram). Second, we compared among women receiving medications indicated for epilepsy the rates of discontinuation and continuation. Continuation of treatment was defined as the presence of at least one dispensation of an ASM in each trimester of pregnancy (the ASM could be different), whereas discontinuation was defined as the absence of dispensation in the first, second, or third trimester of pregnancy. The first trimester of pregnancy (T1) was defined as the period from the LMP date to 13 weeks of amenorrhea + 6 days, the second trimester (T2) from 14 to 27 weeks of amenorrhea + 6 days, and the third trimester (T3) from 28 weeks of amenorrhea to the end of the pregnancy. We also examined the number of concomitant ASM dispensations and the dosage, using the World Health Organization defined daily dose (DDD) calculation.^13^ We therefore calculated the average number of DDDs per month for each trimester and described the rate of women receiving polytherapy. Third, we described and compared maternal and neonatal outcomes between the periods "before" and "after" the pictogram. Maternal outcomes included the rate of hospitalizations for epilepsy (ICD‐10 codes: “epilepsy and recurrent seizures” G40, “status epilepticus” G41, or “other and unspecified convulsions” R56. 8, as main reason for admission) and/or for falls (ICD‐10 codes: W18 and W19), the mean total number of days of hospital stays (all causes combined), and the rate of intensive care unit admission during pregnancy and in the year postpartum. Neonatal outcomes included the rate of stillbirths, the rate of prematurity (<37 weeks of amenorrhea), and the rate of medical termination of pregnancy.

We conducted a subgroup analysis of the discontinuation and continuation of ASM, focused on women with a high likelihood of having epilepsy. These medications may have been prescribed for indications other than epilepsy, particularly for neuropathic pain, migraine, and mood disorders. To increase the likelihood that the study group had epilepsy as the reason for being prescribed ASM, we used an algorithm developed by the French National Health Insurance Fund.^14^ We selected women who had a code indicating epilepsy during hospitalization (PMSI database) or with epilepsy registered in the SNDS as a long‐term condition (ICD‐10 codes: G40 and G41). Among women without a specific epilepsy diagnosis, we excluded the following profiles:

- Monotherapy and no prescription from a neurologist during the study period for the following medications: carbamazepine, oxcarbazepine, valproic acid, lamotrigine, or topiramate.
- Monotherapy or combination therapy with clonazepam, gabapentin, and/or pregabalin (and no other ASMs) during the study period.
- Monotherapy with carbamazepine, oxcarbazepine, or phenytoin and at least three dispensing dates for a medication used for neuropathic pain (capsaicin, duloxetine, amitriptyline, maprotiline, venlafaxine, clomipramine, imipramine, trimipramine) during the study period.
- Monotherapy with topiramate AND ≥3 dispensing dates for a medication used for migraine (selective 5HT1 receptor agonists [ATC class N02CC], ergot alkaloids [ATC class N02CA], pizotifen, erenumab, oxetorone) during the study period.

In addition, a sensitivity analysis was performed by excluding the COVID‐19 pandemic period (i.e., pregnancies with a delivery date after January 2020).

Statistical analyses were performed using SAS Enterprise Guide version 8.3 software (SAS Institute).

## 3 RESULTS

During the “before pictogram” period, 14 732 women of 2 213 081 (.7%) had been dispensed at least twice with a medication indicated for epilepsy in the year before the start of their pregnancy, and 14 348 of 2 080 919 (.7%) during the “after pictogram” period.

The average age of the women at the time of their pregnancy was 31.5 years (±5.3 [16–52]) in the “before pictogram” period and 31.7 years (±5.3 [16–55]) in the “after pictogram” period.

### 3.1 Antiseizure medications

Table 1 shows the list of medications indicated for epilepsy dispensed during pregnancy in both periods. These medications may have been prescribed for epilepsy or other indications. The most frequently dispensed ASMs during pregnancy were lamotrigine, levetiracetam, clobazam, and pregabalin. Clobazam is most often prescribed at the end of pregnancy and in combination with lamotrigine. We observed changes in this ranking between the two studied periods, with an increase in the rate of women exposed to lamotrigine (29.9% during the “before pictogram” period vs. 31.5% during the “after pictogram” period, *p* = .002), levetiracetam (10.9% vs. 14.5%, *p* < .0001), and pregabalin (5.5% vs. 7.2%, *p* < .0001). Conversely, we observed a decrease in the rate of women exposed to valproic acid (5.4% vs. 1.3%, *p* < .0001), carbamazepine (3.6% vs. 2.6%, *p* < .0001), and topiramate (2.9% vs. 2.4%, *p* = .002) between the two periods.

The mean number of different ASMs dispensed before and/or during pregnancy was similar in both periods (1.5 ± .8 [1–8]). On average, 10.6 (±7.9 [2–64], *n* = 14 732) different dates of dispensation of ASMs were recorded before and/or during pregnancy in the “before pictogram” period and 11.1 (±8.2 [2–116], *n* = 14 348) in the “after pictogram” period.

In approximately two thirds of cases, ASMs were prescribed during pregnancy by a general practitioner (67.2% vs. 58.2%) and in almost one quarter by a neurologist (22.8% vs. 28.0%). During the “after pictogram” period, we observed an increase in ASM prescriptions by neurologists (*p* < .0001) and a corresponding decrease in prescriptions by general practitioners (*p* < .0001).

### 3.2 Discontinuation and continuation of ASMs

Table 2 shows the rates of women who stopped and who continued their antiseizure treatment in the total population (all indications included) and in the subgroup of “epileptic” women. In the total population, the number of women continuing their treatment increased by 2.7% during pregnancy (i.e., at least one prescription in each trimester; 37.6% vs. 40.3%, *p* < .0001), whereas the number of women stopping treatment before or during pregnancy decreased by 2.7% in the "after pictogram" period (59.4% vs. 56.7%, *p* < .0001). In the subgroup of “epileptic” women, the percentage of women who continued their treatment was higher than in the total population. There was no change in treatment discontinuation and continuation between the two periods in this subgroup.

**TABLE 2:** Discontinuation and continuation of antiseizure medications in the total population of women who received these medications before pregnancy^a^ and in the subgroup of “epileptic” women; *N* = 29 080.

	Total population			“Epileptic” population		
	"Before pictogram" period, *n* = 14 732	"After pictogram" period, *n* = 14 348	*p*	"Before pictogram" period, *n* = 7837	"After pictogram" period, *n* = 8065	*p*
	Women, *n* (%)			Women, *n* (%)		
Continuation of the anti‐seizure treatment	5536 (37.6%)	5782 (40.3%)	**<.0001**	4809 (61.4%)	4879 (60.5%)	.26
Discontinuation of the anti‐seizure treatment	8746 (59.4%)	8133 (56.7%)	**<.0001**	2688 (34.3%)	2861 (35.5%)	.12
Y^−1^	6617 (44.9%)	6129 (42.7%)		1812 (23.1%)	1968 (24.4%)	
Y^−1^–T1	1689 (11.5%)	1564 (10.9%)		589 (7.5%)	631 (7.8%)	
Y^−1^–T1‐T2	440 (3.0%)	440 (3.1%)		287 (3.7%)	262 (3.2%)	
Other profiles	450 (3.0%)	433 (3.0%)	.85	340 (4.3%)	325 (4.0%)	.33
Y^−1^–T1‐T3	145 (1.0%)	128 (.9%)		113 (1.4%)	94 (1.2%)	
Y^−1^–T2	97 (.6%)	116 (.8%)		67 (.8%)	88 (1.1%)	
Y^−1^–T2‐T3	130 (.9%)	121 (.8%)		109 (1.4%)	102 (1.3%)	
Y^−1^–T3	78 (.5%)	68 (.5%)		51 (.6%)	41 (.5%)	

*Note*: Boldface indicates statistical significance. Abbreviations: T1, first trimester; T2, second trimester; T3, third trimester; Y^−1^, 1 year before last menstrual period.

^a^ At least two dispensations in the year before the start of the pregnancy.

The increase in continuing treatment without stopping was particularly marked among women taking lamotrigine (37.7% vs. 40.0%, *p* = .01), levetiracetam (20.8% vs. 28.2%, *p* < .0001), and pregabalin (1.3% vs. 3.6%, *p* < .0001). A reduction in treatment discontinuation was observed in women treated with pregabalin (prepregnancy discontinuation: 53.6% vs. 49.5%, *p* = .0001; first trimester discontinuation: 10.7% vs. 9.3%, *p* = .03) and valproate (prepregnancy discontinuation: 32.6% vs. 24.1%, *p* < .0001). There were therefore far fewer women taking valproate in the "after pictogram" period, and these women were less likely to stop their treatment during pregnancy.

### 3.3 Intensity of exposure

A total of 14.9% of women had concomitant prescriptions for several ASMs, and this rate did not change between the “before” and “after” periods (15.0% vs. 14.8%, *p* = .54). The rate of women switching from polytherapy before pregnancy to monotherapy during pregnancy fell slightly between the two periods (3.4% vs. 2.9%, *p* = .01).

Table 3 shows the average number of DDDs per month of medications indicated for epilepsy per trimester and per "before"/"after" period. For both periods, there was a gradual decrease in the average number of DDDs per month for ASMs before the start of pregnancy, followed by a gradual increase of DDDs during pregnancy. The average number of DDDs per month (before and during pregnancy) increased very slightly in the period after the pictogram was introduced. This increase in doses was particularly notable for lamotrigine and pregabalin.

**TABLE 3:** Mean number of DDDs per month of antiseizure medications per pregnancy trimester in women who received these medications before pregnancy^a^; *N* = 29 080.

Period	DDDs per month, mean ± SD		
	"Before pictogram" period	"After pictogram" period	*p*
Before pregnancy	*n* = 14 732	*n* = 14 348	
T‐4	*n* = 10 454	*n* = 10 360	.**001**
	42.5 ± 68.3	45.8 ± 78.1	
T‐3	*n* = 11 040	*n* = 10 861	**<.0001**
	31.7 ± 53.5	36.0 ± 90.6	
T‐2	*n* = 11 006	*n* = 10 868	**<.0001**
	31.0 ± 47.5	34.8 ± 65.3	
T‐1	*n* = 10 073	*n* = 10 128	**<.0001**
	29.3 ± 36.6	33.4 ± 49.5	
During pregnancy	*n* = 8115	*n* = 8219	
T1	*n* = 7810	*n* = 7914	**<.0001**
	31.1 ± 36.4	35.6 ± 52.5	
T2	*n* = 6203	*n* = 6459	**<.0001**
	36.7 ± 38.1	40.9 ± 51.1	
T3	*n* = 5834	*n* = 6034	**<.0001**
	40.2 ± 41.4	44.8 ± 54.4	

*Note*: Boldface indicates statistical significance.

Abbreviations: DDD, defined daily dose; LMP, last menstrual period; T‐1, the trimester before the date of LMP; T‐2, between 6 months and 3 months before the date of LMP; T‐3, between 9 months and 6 months before the date of LMP; T‐4, between 1 year and 9 months before the date of LMP; T1, 1st trimester of pregnancy; T2, 2nd trimester of pregnancy; T3, 3rd trimester of pregnancy.

^a^ At least two dispensations in the year before the start of the pregnancy.

### 3.4 Maternal outcomes

The rate of women hospitalized for epilepsy during pregnancy and/or within 1 year after the pregnancy outcome was similar throughout the study period (546 women, i.e., 3.7% in the “before pictogram” period and 498 women, i.e., 3.5% in the “after pictogram” period; *p* = .28). The average number of days spent in hospital during pregnancy (all diagnoses combined), including the stay for delivery, was 4.7 ± 11.4 days in the “before pictogram” period and 4.6 ± 11.3 days in the “after pictogram” period (*p* = .19). The average number of hospitalization days during the postpartum period was slightly higher in the “before” period (8.7 ± 20.5 days) than in the “after” period (8.2 ± 19.4 days, *p* < .0001).

The rate of women admitted to the intensive care unit did not differ between the “before” and “after” periods (2.3% during pregnancy vs. 2.8% and 2.5% in the 1 year after the delivery for the two periods).

Overall, 27 women (.1%) died during their hospital stay or in the postpartum period (“before pictogram”: 12 women [.1%]; “after pictogram”: 15 women [.1%]; *p* = .52). The causes of death were unknown.

### 3.5 Neonatal outcome

The rate of stillbirths increased slightly in the period following the introduction of the pictogram, with a rate of .8% (i.e., 118 stillbirths) compared to the “before” period with a rate of .6% (i.e., 82 stillbirths, *p* = .02). There was no significant difference in the rate of medical termination of pregnancy between the two periods (.5% vs. .4%, *p* = .20) nor in the rate of prematurity (8.8% vs. 9.1%, *p* = .47).

### 3.6 Sensitivity analysis

The results of the sensitivity analysis, which was carried out after the exclusion of the COVID‐19 period, are comparable to those of the main analysis in terms of the changes observed in the rates of treatment discontinuation and continuation, as well as in the medications used during the two study periods.

## 4 DISCUSSION

According to the results of this study, the introduction of the pregnancy pictogram in France in 2017 has not led to a significant change in the prescription and dispensation practices for medications indicated for epilepsy during pregnancy.

### 4.1 Impact of the pregnancy pictogram on practices

The introduction of the pregnancy pictogram on medication packaging in 2017 in France was not associated with any negative changes in the prescription and dispensation practices for ASMs during pregnancy. The same was true for a subgroup of women highly likely to receive these medications due to epilepsy. We were particularly interested in these epileptic women because in this population, like some others with chronic conditions (asthma, depression, etc.), there is a high maternal risk if treatment is stopped. Ultimately, our study did not show an increase in treatment discontinuation in the period following the addition of the pictogram to medication packaging. We observed a slight increase in the proportion of pregnant women exposed to the lowest risk ASMs, lamotrigine and levetiracetam, and a parallel decrease in the proportion of women exposed to the highest risk ASMs, topiramate and, in particular, valproate. Similar trends have been observed in other international studies, with an increase in prescriptions for levetiracetam and lamotrigine and a decrease in those for valproate and topiramate.^15^, ^16^, ^17^ The significant reduction in valproate exposure may also be linked to the alerts and measures introduced by French health authorities. New measures were introduced in 2018, including a contraindication for valproate use in girls, women of childbearing age, and pregnant women, unless there are no alternative options (for epileptic women).^18^ In France, the harmful effects of valproate on the neurodevelopment of children exposed to it in utero received considerable media coverage around 2020 due to patient complaints. The study also showed that, in the postpictogram period, prescriptions for epilepsy medications were slightly more likely to come from specialists. This may also be linked to the requirement for an initial prescription of valproate to be made by a specialist, such as a neurologist or psychiatrist. This requirement was introduced in France in 2017.^19^

Many institutions and health care professionals feared that the pictogram would lead pregnant women to stop taking their medication without medical advice or health care professionals to stop it. However, the results show that the rate of pregnant women who interrupted or continued their antiepileptic treatment changed little between the periods before and after the introduction of the pictogram. There was a slight decrease in the number of discontinuations and a slight increase in the number of continuing treatments throughout pregnancy. The number of DDDs per month even increased slightly between the periods “before” and “after” the introduction of the pictogram. This may be related to the finding that more women switched to lamotrigine in the period “after the pictogram.” The lamotrigine dosages used in women previously treated with valproate are certainly often higher than the DDD. Furthermore, a study has shown that the ratio of prescribed dose to DDD was higher with lamotrigine than with valproate.^20^

To our knowledge, no study on the impact of the pictogram introduced in France in 2017 has yet been published. A survey was conducted in two hospitals to examine women's perceptions and comprehension of the pictogram, with a particular focus on pregnant women and those of childbearing age. The results revealed poor and inaccurate comprehension of the pictogram's meaning. Furthermore, nearly 80% of pregnant women stated that they would immediately stop taking medication with the pictogram on its packaging without seeking medical advice.^21^ The pregnancy pictogram is a French initiative. Apart from a pictogram on the packaging of medications containing valproate or retinoids in the United States and the Netherlands, other countries have not used this means to warn of the risks of medications during pregnancy. Consequently, the impact of this visual means of communication has not been evaluated in other countries. Our study is therefore the first to examine the possible consequences of a pregnancy pictogram.

A few studies on medications other than ASMs have assessed patients' understanding of pictograms on medication packaging.^22^, ^23^ However, none of these studies has evaluated the actual impact on medication use practices. One study examined the effect of the “danger to driving” pictogram by comparing the number of road accidents associated with exposure to benzodiazepine and related hypnotic agents before and after the pictogram was introduced on their packaging in 2008.^24^ However, the risk of road accidents associated with benzodiazepines and related medications increased slightly over time, suggesting that the pictogram was ineffective.

### 4.2 Strengths and limitations of the study

SNDS data have the advantage of national and historical coverage. Prescription and dispensation data from the French National Health Insurance Service provides reliable and exhaustive information on reimbursable medications dispensed during pregnancy and avoids the memory bias that can occur when medication intake is collected via questionnaires. Using the SNDS as the data source allowed us to include a large number of pregnant women in this study, which is another one of its strengths.

Among the limitations of the SNDS, it should be noted that the health insurance scheme does not record medications dispensed in hospitals or nonreimbursable medications. This should not affect our results, because all ASMs are reimbursable and dispensed in community pharmacies. Furthermore, the dispensation of a medication does not necessarily mean that it has been consumed. To compensate for the lack of data on compliance, we used DDDs, which enabled us to limit this bias and to assess the average dose of medication ingested per month. Finally, the results of this study should not be generalized to all medications, but only to the impact of the pictogram on prescription and dispensation practices for medications indicated for epilepsy. This choice was made because stopping the use of medication for epilepsy can lead to serious complications for pregnant women and their pregnancy.^3^, ^4^, ^5^

As ASMs can be prescribed for conditions other than epilepsy, more than half of our study population is unlikely to have epilepsy. The algorithm we used to increase the specificity of epilepsy diagnosis may have excluded some women with epilepsy due to the restrictions applied. However, these limitations were identical in both the “before pictogram” and “after pictogram” periods and should therefore not affect the interpretation of the results.

Finally, our analyses were conducted among the whole population of pregnant women who used ASM before pregnancy; we cannot exclude that there may be unfavorable patterns of medication variation between the "before" and "after" periods in certain subgroups of women, such as those with psychosocial vulnerability or those with previous adverse pregnancy outcomes. As we could not explore those in this analysis, further research is needed to explore these aspects.

## 5 CONCLUSIONS

Since the pregnancy pictogram appeared on medication packaging in France, there has been little change in the prescription and dispensation of medications for epilepsy during pregnancy. The results of this study did not show an increase in the discontinuation of ASMs by pregnant women. Our findings suggest that there has been an improvement in the care provided to women treated for epilepsy, with a decrease in prescriptions for teratogenic ASMs and a slightly more frequent switch from polytherapy to monotherapy. However, it is not possible to attribute this improvement solely to the pictogram, as information campaigns made in France during the same period are a complementary means of raising awareness of the importance of optimizing the treatment of epilepsy during pregnancy.

## AUTHOR CONTRIBUTIONS

Catherine Deneux‐Tharaux, Monica Saucedo, Christine Damase‐Michel, and Isabelle Lacroix obtained the funding. Mélanie Araujo and Laurane Delteil were involved in acquisition of data. Mélanie Araujo, Laurane Delteil, Christine Damase‐Michel, Monica Saucedo, Catherine Deneux‐Tharaux, and Isabelle Lacroix were involved in conception and design of the study. All authors were involved in analysis of data, interpretation of data, and drafting the article.

## FUNDING INFORMATION

This study was funded by EPI‐PHARE (EC 2021‐07; https://www.epi‐phare.fr). The content of this publication is solely the responsibility of the authors and does not necessarily reflect the views of the relevant health authorities.

## CONFLICT OF INTEREST STATEMENT

S.B. has received consultancy fees from Angelini, Eisai, and UCB outside the submitted work. The other authors have no conflict of interest to declare. We confirm that we have read the Journal's position on issues involved in ethical publication and affirm that this report is consistent with those guidelines.

## ETHICS STATEMENT

The SNDS was approved by the French Data Protection Agency. This study was performed on pseudonymized patient data.

## Data Availability

All datasets used during the current study have been extracted from the French National Health Data System (SNDS) upon specific approval and are not publicly available, as access to SNDS data is subject to prior training and authorization.
